# Silencing of Ribosomal Protein S9 Elicits a Multitude of Cellular Responses Inhibiting the Growth of Cancer Cells Subsequent to p53 Activation

**DOI:** 10.1371/journal.pone.0009578

**Published:** 2010-03-08

**Authors:** Mikael S. Lindström, Monica Nistér

**Affiliations:** Department of Oncology-Pathology, Karolinska Institutet, Stockholm, Sweden; Health Canada, Canada

## Abstract

**Background:**

Disruption of the nucleolus often leads to activation of the p53 tumor suppressor pathway through inhibition of MDM2 that is mediated by a limited set of ribosomal proteins including RPL11 and RPL5. The effects of ribosomal protein loss in cultured mammalian cells have not been thoroughly investigated. Here we characterize the cellular stress response caused by depletion of ribosomal protein S9 (RPS9).

**Methodology/Principal Findings:**

Depletion of RPS9 impaired production of 18S ribosomal RNA and induced p53 activity. It promoted p53-dependent morphological differentiation of U343MGa Cl2:6 glioma cells as evidenced by intensified expression of glial fibrillary acidic protein and profound changes in cell shape. U2OS osteosarcoma cells displayed a limited senescence response with increased expression of DNA damage response markers, whereas HeLa cervical carcinoma cells underwent cell death by apoptosis. Knockdown of RPL11 impaired p53-dependent phenotypes in the different RPS9 depleted cell cultures. Importantly, knockdown of RPS9 or RPL11 also markedly inhibited cell proliferation through p53-independent mechanisms. RPL11 binding to MDM2 was retained despite decreased levels of RPL11 protein following nucleolar stress. In these settings, RPL11 was critical for maintaining p53 protein stability but was not strictly required for p53 protein synthesis.

**Conclusions:**

p53 plays an important role in the initial restriction of cell proliferation that occurs in response to decreased level of RPS9. Our results do not exclude the possibility that other nucleolar stress sensing molecules act upstream or in parallel to RPL11 to activate p53. Inhibiting the expression of certain ribosomal proteins, such as RPS9, could be one efficient way to reinitiate differentiation processes or to induce senescence or apoptosis in rapidly proliferating tumor cells.

## Introduction

Ribosome biogenesis is a highly complex process in which hundreds of different proteins co-operate in ribosomal RNA (rRNA) synthesis, processing, ribosome subunit assembly and transport [1]. Nucleoli are the actual sites for ribosome biogenesis but these structures have also been implicated in other cellular processes such as control of the cell cycle, viral replication, mitogenic signaling, nuclear export of p53, and stem cell differentiation, reviewed in references [1]–[4]. Over the last decade, the connection between the tumor suppressor protein p53, the nucleolus, and ribosome biogenesis has become well established. Expression of dominant negative mutants of the nucleolar protein Bop1 resulted in defects in rRNA processing triggering p53 induced cell cycle arrest [5]. p53 often becomes activated after silencing of nucleolar or ribosomal proteins (r-proteins), and examples include RPL23 [6], RPS6 [7], nucleostemin, [8] and TIF1A [9]. Moreover, inhibitors of rRNA synthesis or processing such as actinomycin D and 5-fluorouracil also activate p53 [10]. Collectively these conditions are referred to as nucleolar or ribosomal stress. Mounting evidence from a number of mouse models proves the existence of this p53-dependent checkpoint *in vivo*. One mouse model revealed that ribosome biogenesis is impaired after conditional deletion of one allele of *Rps6* [11]. Interestingly, the embryonic lethality that occurred during gastrulation in these *Rps6^+/−^* embryos was caused by a p53-dependent checkpoint rather than a general decrease in translational capacity [11]. Rpl22 deficiency selectively arrested development of alpha/beta-lineage T cells, a response mediated by activation of p53 [12]. In turn, p53 loss restored the development of Rpl22-deficient thymocytes.

Mutations have been found in different r-proteins in patients suffering from a syndrome known as Diamond-Blackfan anemia (DBA, MIM #105650), characterized by defective erythropoiesis, congenital anomalies and an increased risk of cancer [13], [14]. The gene *RPS19* is frequently mutated in DBA [15], but mutations have also been found in *RPS24*, *RPS17*, *RPL35A*, *RPL5*, *RPL11*, and *RPS7* [16], [17]. Animal models of DBA have been created in mice and zebrafish [18], [19]. It was found that loss of *Rps19* is embryonic lethal [20]. *Rps19* deficiency impairs ribosomal biogenesis and activates p53 in zebrafish, and the suppression of p53 and deltaNp63 can alleviate the Rps19-deficient phenotype in this model [18]. Loss of other r-proteins in zebrafish, such as Rps8, Rps11, and Rps18 also triggered a p53 response [18]. The role of p53 in DBA remains unclear but it is notable that mice bearing mutations in *RpS19* and *Rps20* have low erythrocyte counts that can be restored by p53 deficiency [21].

How is p53 activated following nucleolar stress? Several groups have shown that r-proteins (RPL11, RPL5, RPL23 and RPS7) are released from the nucleolus during stress and then bind MDM2 thereby activating p53 [6], [22]–[29]. It was subsequently found that increased translation of 5′ terminal oligopyrimidine (TOP) mRNAs, including that of *RPL11*, occurs in response to disrupted 40S ribosomal subunit biogenesis thereby leading to an increase in RPL11 production [30]. Indeed, following loss of RPS6 or RPL24 the p53 tumor suppressor is activated in a manner dependent on RPL11 [30], [31]. While decreased expression of some r-proteins can activate p53 as part of a cellular stress response, it is also evident that some other r-proteins have direct and more specific roles in *p53* mRNA translation. Haploinsufficiency of a subset of r-proteins in zebrafish is linked to the development of malignant peripheral nerve sheath tumors [32]. Interestingly, these nerve sheath tumors fail to produce p53 protein, despite presence of *p53* mRNA [33]. Moreover, RPL26 plays a role in enhancing *p53* mRNA translation during the DNA damage response by direct interactions with both MDM2 protein and *p53* mRNA [34]. Hence, r-proteins have more dynamic roles in regulating the p53 tumor suppressor pathway than was previously thought, reviewed in [35]–[37].

Recently, RPS9 was identified as a novel B23 (NPM/nucleophosmin) interacting protein [38]. Decreased levels of RPS9 were associated with accumulation of cells in G_1/_(G_0_) and p21 induction in U2OS osteosarcoma cells [38]. Here we set out to further investigate the different cellular stress responses caused by loss of RPS9. Depletion of RPS9 provoked a rapid loss of the nucleolar protein pool, impaired production of mature 18S ribosomal RNA and activation of the p53 tumor suppressor pathway. We found that the combination of a defective ribosome biogenesis pathway and p53 activation resulted in unexpectedly strong anti-proliferative responses in human tumor cell lines in that they underwent senescence, differentiation, or apoptosis following depletion of RPS9.

## Results

### Rapid Depletion of Nucleolar RPS9 Following siRNA Transfection

**Figure 1:**
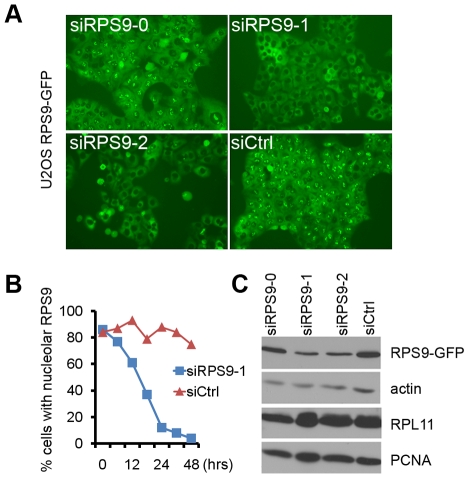
Nucleolar and cytoplasmic localization of RPS9-GFP. (A) Efficiency of RPS9-GFP knockdown in U2OS cells that stably express RPS9-GFP when using three different siRNAs targeting human RPS9. Photos of cells expressing RPS9-GFP were taken after 48 hours. (B) Percentage of U2OS cells expressing detectable RPS9-GFP in the nucleolus at various time points after transfection of RPS9-1 siRNA or siCtrl. (C) Equal number of cells from experiment in (A) was collected and whole cell extracts prepared in buffer containing 2% SDS. Protein lysates were separated by SDS-PAGE and immunoblotted with antibodies specific for EGFP, PCNA, β-actin, and RPL11 as indicated in the figure.

To fuse r-proteins with GFP has proved a useful tool to better visualize the localization and turnover of r-proteins in human cells [39], [40]. U2OS cells expressing RPS9-GFP had been previously established [38]. In these cells the RPS9-GFP fusion protein was localized to the nucleolus and the cytoplasm. To better understand the dynamics of RPS9 in relation to knockdown phenotypes, we used RPS9-GFP U2OS cells combined with RNA interference to visualize knockdown of RPS9 in living cells (Figure 1A). Transfection of U2OS cells with RPS9 siRNA indicated a rapid turnover of nucleolar RPS9-GFP. Already 24 hours after transfection a majority of the cells showed a striking loss of nucleolar RPS9-GFP (Figure 1B). At 72 hours post-transfection, most cells had a GFP signal detectable only in the cytoplasm. Using immunoblotting RPS9-GFP was detected as a single band of the expected size that was decreased upon siRNA transfection with two different RPS9 siRNAs #1 and #2, whereas knockdown with oligo #0 was ineffective when compared to the control siRNA, siCtrl (Figure 1C). The pool of cytoplasmic RPS9-GFP protein remained for several days in agreement with the known stability of mammalian cytoplasmic ribosomes. We have been unable to completely deplete cells of cytoplasmic GFP reactivity which could be due to the fact that silencing with siRNA is not 100%, or that a total loss of RPS9 is not compatible with sustained cell proliferation. To further confirm the cellular distribution of RPS9, U2OS cells were stained with an antibody raised against human RPS9, as described [38], and an identical nucleolar and cytoplasmic staining was observed. The nucleolar pool of endogenous RPS9 could be rapidly and efficiently silenced in U2OS cells similar to that of RPS9-GFP (Figure S1A), and this was confirmed by immunoblotting (Figure S1B). In summary, we obtained a complete and specific depletion of nucleolar RPS9 using siRNA.

### Induction of a Limited Senescence Response in U2OS Cells

Disruption of the nucleolus is often seen following exposure of cells to a variety of chemical and physical agents that inhibit transcription or rRNA processing [10]. A close inspection of nucleoli revealed that they remained intact in response to treatment with siRPS9, in agreement with studies on RPS6 [11], [30]. But, we noted that nucleoli in RPS9 depleted U2OS cells were more contrasted, round, and phase-dense than in siCtrl treated cells indicating a rearrangement of the nucleolar structure (Figure S2A). Nucleolar dense fibrillar centers were in a few cells re-localized to the periphery of the nucleolus as revealed by staining for fibrillarin, a marker of the dense fibrillar compartment of the nucleolus. These nucleoli resembled the enlarged nucleoli observed in U2OS cells depleted of nucleostemin [8], a nucleolar protein involved in ribosome biogenesis [41].

**Figure 2:**
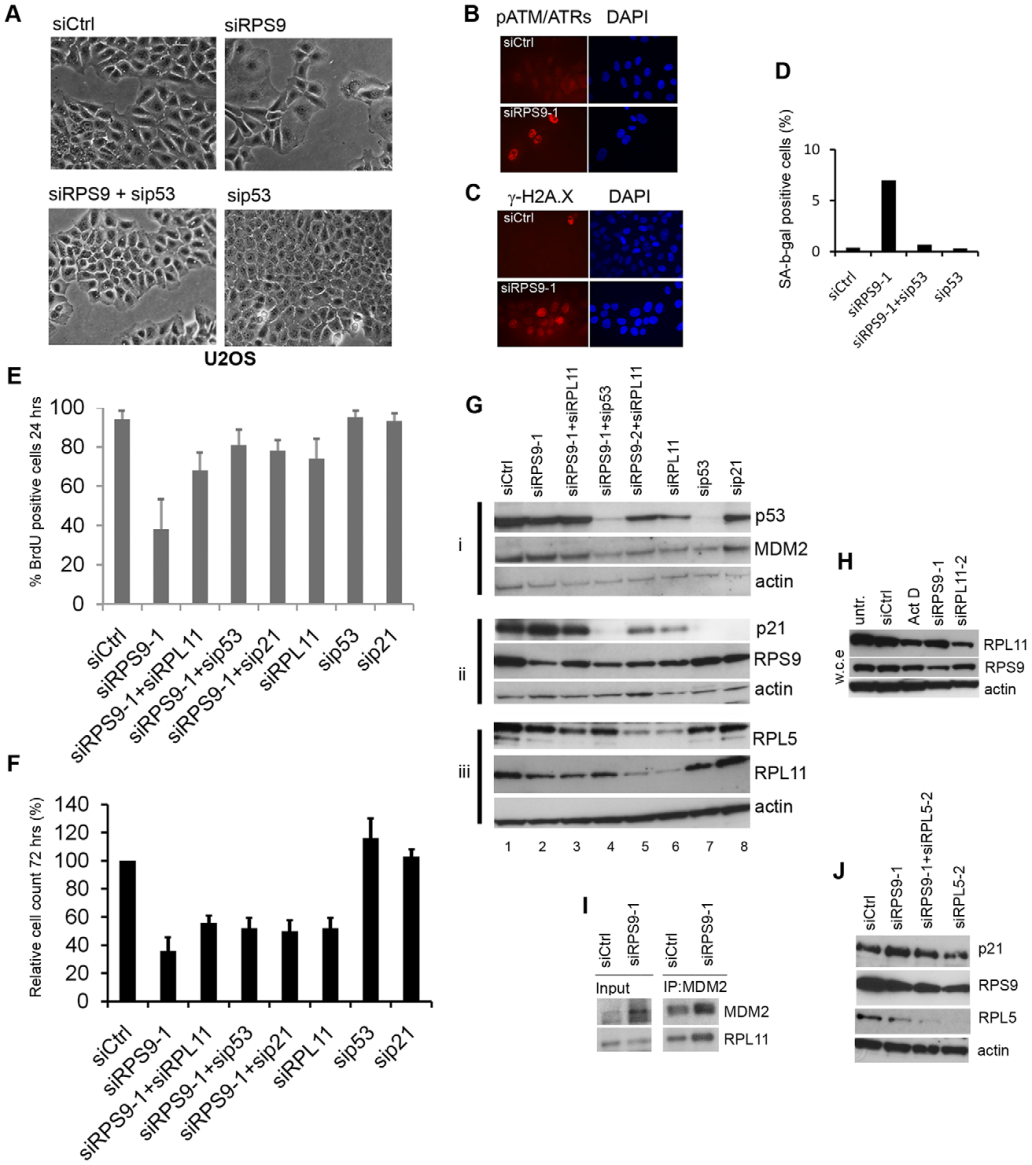
Silencing of RPS9 induces a limited senescence response in U2OS cells. (A) Phase contrast images reveals morphology of human U2OS osteosarcoma cells transfected with siRNA targeting RPS9 or p53. (B–C) Increased expression of DNA damage and replication stress markers in U2OS cells depleted of RPS9 and analyzed 72 hours after siRNA transfection. U2OS cells were fixed and stained with an antibody towards γ-H2AX and a phospho-ATM/ATR substrate antibody. (D) Quantification of SA-β-gal positive U2OS cells in cultures depleted of RPS9. Shown is one representative experiment out of two. (E) Cells were transfected with siCtrl, RPS9, RPL11, p53, or p21 siRNA as indicated. The cells were incubated with BrdU at 24 hour post transfection for another 24 hours and the cells were then fixed and stained with anti-BrdU antibodies and the average of the BrdU-positive cells is shown (%). (F) Co-depletion of RPL11, p53 or p21 partially impaired RPS9 knockdown-mediated inhibition of cell proliferation. U2OS cells were transfected with siCtrl, siRPS9, sip53, sip21 or siRPL11 in combinations as indicated. Final concentration of siRNA was 100 nM in each case and compensation was made with siCtrl. Cells were counted 72 hours post-transfection and shown is mean and SEM from three different experiments performed in triplicate at independent occasions and normalized to siCtrl set to 100%. (G) Co-knockdown of RPL11 attenuated induction of p21 and MDM2 proteins caused by knockdown of RPS9. U2OS cells were transfected with combinations of siRNA as indicated. Cell lysates were subjected to immunoblotting and the expression of p53, MDM2, p21, RPL11, RPL5 and RPS9 was determined as indicated. The protein lysate from each sample was split for three different blots and each probed with actin as a loading control. (H) U2OS cells were transfected with siRPS9-1 or siRPL11-2 for 48 hours or treated with 5 nM actinomycin D for 18 hours after which the cells were harvested directly into 2% SDS containing lysis buffer. Cell lysates were subjected to immunoblotting using antibodies towards RPL11 or RPS9 (w.c.e =  whole cell extract). (I) Co-immunoprecipitation of MDM2 and RPL11 in U2OS cells transfected with siRNA targeting RPS9. MDM2 was immunoprecipitated with N20 antibody followed by detection with SMP14 monoclonal or a monoclonal towards RPL11. Note, that different exposure times were used for RPL11 IP and input blots. (J) U2OS cells were transfected with siRPS9-1 alone or in combination with siRPL5-2 for 72 hours, after which cells were harvested and the level of p21 as a read-out for p53 activity was analyzed by immunoblotting.

Knockdown of RPS9 induced accumulation of cells in G_1_ and increased the levels of p21 in U2OS cells [38]. A more careful examination of the cells revealed that a fraction displayed a rather “severe” phenotype resembling senescence with a grossly expanding cytoplasm leading us to further investigate this particular response (Figure 2A). Depletion of RPS9 induced an increase in the number of γ-H2A.X positive cells and cells with nuclear foci of phospho-ATM/ATR substrate proteins (Figure 2B and C). Analysis revealed an increase of senescence associated β-galactosidase–positive (SA-β-gal) U2OS cells from 0.4% positive cells in siCtrl cells, to 7% in siRPS9 transfected cells (Figure 2D).

To formally demonstrate the role of p53 in mediating inhibition of U2OS cell proliferation we co-depleted p53 and RPS9 resulting in a reversion of the senescence phenotype (Figure 2A and D). In addition, RPL11, p53 or p21 co-depletion in siRPS9-treated cells increased BrdU incorporation in these cultures at 24 hours post-transfection (Figure 2E), coupled with a rescue of the senescence-like morphology (Figure S3). We wanted to know how this “escape” from G_1_ arrest related to cell growth in the longer run, so we also investigated cell proliferation at a later time point. We found that siRPS9 treated cultures had grown 36% (±9.2) whereas siRPS9+sip53 treated cells had grown 52% (±6.5) compared to siCtrl as evaluated 72 hours post-transfection (Figure 2F). Silencing of RPL11 restored cell count to the level seen in cells co-transfected with siRPS9 and sip53, but it could not restore the cell number to that seen in siCtrl cultures, hence siRPS9+siRPL11 treated cells had grown 56% (±4.4) compared to siCtrl (Figure 2F). This indicated that silencing of RPS9 or RPL11 also induced suppression of cell proliferation in a p53-independent manner. This was confirmed by depleting RPS9 in matched WI38 and WI38-E6 fibroblasts as well as in SAOS2 osteosarcoma cells lacking p53 (Figure S1D).

### Regulation of the p53 Response Triggered by RPS9 Loss in U2OS Cells

The decreased expression of RPS9 in U2OS cells did not markedly change the levels of p53 as shown before [38], but induced an increase in p21 protein that was dependent on p53 (Figure 2G). Induction of p21 was markedly impaired by co-transfecting cells with RPL11 siRNA (Figure 2G). Knockdown of RPL11 using siRPL11-1 resulted in lower levels of p53, MDM2, and in particular p21, also under normal conditions in U2OS cells (Figure 2G, lane 6). Interestingly, depletion of RPS9 led to lower levels of NP40-soluble RPL11 (Figure 2G, lane 2) but the fraction of RPL11 bound to MDM2 remained the same and did not decrease (Figure 2I). We wanted to know if the reduction in RPL11 also could be seen in whole cell extracts and therefore U2OS cells were transfected with siRPS9 or incubated in 5 nM actinomycin D for 18 hours. This concentration of actinomycin D selectively blocks RNA Pol I activity and prevents ribosome biogenesis. We could confirm the results obtained when using the milder lysis buffer also in whole cell extracts thus showing decreased total levels of RPL11 (Figure 2H). Most of the studies in the field have relied upon silencing RPL11 using one particular siRNA so we found it of importance to compare RPL11 with RPL5. We found that depletion of RPL5 could block p21 induction identical to RPL11 in RPS9 depleted cells (Figure 2J). Silencing of RPL11 with morpholinos induces a p53-dependent apoptotic response in zebrafish [42], and we therefore do not want to rule out that RPL11 and RPL5 loss can to some extent induce p53 activity or engage p53 related pathways *in vivo*. We also made another observation from the experiment in figure 2G. First, the knockdown of RPL11 caused a decline in RPL5 protein level to the same extent as for RPL11 (Figure 2G, lane 6). This is presumably because 28S rRNA is not efficiently processed in RPL11 or RPL5 deficient cells [43] leading to a surplus of large subunit r-proteins that are rapidly degraded [44]. It has recently been shown by other groups that depletion of one individual r-protein from one ribosome subunit results in down-regulation of other proteins from the same subunit [45]. This finding might have some implications as to why the regulation of MDM2 by several ribosomal proteins seems to occur in a non-redundant manner, but this cannot be the only explanation because then we would find that knockdown of almost every r-protein can block p53 stabilization which we do not see. In summary, we conclude that RPL11 is required for the efficient induction of p53-dependent responses in RPS9 depleted U2OS cells.

### Is RPL11 Required for p53 mRNA Translation Following Nucleolar Stress?

**Figure 3:**
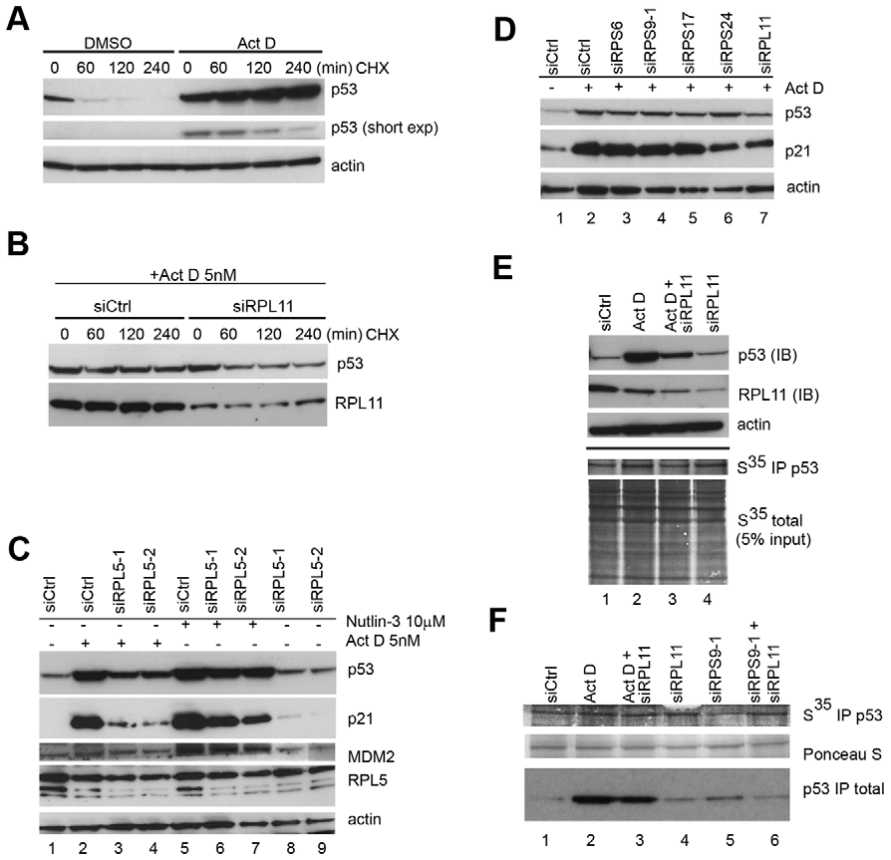
Analysis of p53 mRNA translation in RPL11 depleted cells. (A) U2OS cells were treated with 5 nM actinomycin D for 18 hours or DMSO only followed by a cycloheximide chase (CHX) to estimate p53 protein degradation rates. Cell lysates from each time point were subjected to immunoblotting and the expression of p53 determined in relation to actin levels. (B) U2OS cells were transfected with siCtrl or siRPL11 overnight followed by treatment with actinomycin D for an additional 18 hours. This was followed by CHX chase as above for indicated time. In this case the loading was adjusted to give comparable starting amounts of p53 facilitating analysis of degradation that is more rapid in RPL11 transfected cells. (C) U2OS cells were transfected with siRNAs targeting RPL5 or with siCtrl. Cells were thereafter treated with Nutlin-3 (10 µM) or actinomycin D (5 nM) for 18 hours. The blotting membrane was probed for RPL5, MDM2, p53 and p21. (D) Depletion of ribosomal proteins RPS6, RPS9, RPS17 and RPS24 does not prevent p53 accumulation and p21 induction following actinomycin D treatment but depletion of RPL11 did. U2OS cells were transfected with indicated siRNA for 18 hours followed by treatment with actinomycin D (5 nM) for another 18 hours and expression levels of p53 and p21 were measured by immunoblotting. (E) p53 synthesis in actinomycin D treated cells with or without siRPL11. Total p53 and RPL11 is shown by immunoblotting and newly synthesized p53 was immunoprecipitated with DO1 antibody and visualized by autoradiography. The experiment was carried out 36 hours after siRNA transfection. (F) Evaluation of p53 mRNA translation in U2OS cells depleted of RPL11, RPS9, RPS9+RPL11, or in cells treated with actinomycin D (5 nM) in the presence or absence of RPL11 siRNA. Shown is the amount of newly synthesized p53 during 15 minutes relative to the total amount of p53, and compared to overall protein level as detected with Ponceau S. The experiment was carried out 36 hours after siRNA transfection.

How does RPL11 control p53? Binding of RPL11 and RPL5 directly to MDM2 protein leads to stabilization of p53 via inhibition of MDM2 E3 ubiqutin ligase activity [46]. However, other reports also indicate critical roles for r-proteins in *p53* mRNA translation [33], [47]. Moreover, the significance of the interaction between MDM2 and RPL11/RPL5 in terms of possible effects on *p53* mRNA translation has remained somewhat unclear. This could be relevant to investigate since MDM2 can boost *p53* mRNA translation as has been described to occur in some settings [48], [49]. Indeed, it was suggested that interactions between RPL11/RPL5 and MDM2 may serve a dual purpose to inhibit MDM2 E3 ligase activity, and to promote *p53* mRNA translation at the same time [48], [49]. On the other hand, MDM2 binds to RPL26 and inhibits RPL26 mediated stimulation of p53 mRNA translation [15]. Hence, we decided to re-visit p53 half-life and degradation and to investigate *p53* mRNA translation following nucleolar stress in U2OS cells with and without RPL11. We first carried out a p53 protein half-life assay to confirm the increased stability of p53 after a low dose actinomycin D exposure to induce nucleolar stress. Indeed, a robust increase in the amount and stability of p53 protein was seen, and essentially very little, if any, degradation of p53 occurred in actinomycin D treated cells during the four hour chase (Figure 3A). In contrast, the bulk of p53 protein in DMSO treated control cells was degraded after less than one hour in cycloheximide (Figure 3A). We then transfected U2OS cells with control or siRPL11 for 24 hours and next treated all transfectants with 5 nM actinomycin D overnight followed by a CHX chase. Loading was adjusted to give a comparable starting amount of p53 to facilitate a direct comparison. p53 was clearly more unstable in RPL11 depleted U2OS cells (Figure 3B). Residual stable p53 in siRPL11 samples at later time points probably stems from a population of cells that have responded to actinomycin D but that have not been transfected with siRPL11. We next reasoned that small molecules that disrupt p53-MDM2 binding, block MDM2 transcription or prevent p53 degradation by the proteasome thereby causing p53 accumulation by “default” would act independently of any ribosomal protein-MDM2 interaction. We therefore treated U2OS cells with Nutlin-3. The Nutlin-3 compound disrupts the p53-MDM2 interaction but does not prevent the binding of MDM2 to p53 mRNA [48], [49]. We transfected cells with two different RPL5 siRNA oligos overnight followed by treatment of cells with 5 nM actinomycin D or 10 µM Nutlin-3 for an additional 18 hours (Figure 3C). Each of the two RPL5 siRNAs could efficiently inhibit p53 accumulation following 5 nM actinomycin D, and in particular the induction of p21 was decreased (Figure 3C, lane 3 and 4). In contrast, the effect on p53 and p21 protein levels when we depleted RPL5 in the presence of Nutlin-3 was marginal when normalized to actin. Therefore RPL5 was not required for stabilization of p53, as well as induction of MDM2 and p21 proteins, in Nutlin-3 treated cells. We next wanted to see if other small subunit r-proteins including RPS9 are required for p53 stabilization after actinomycin D treatment. We depleted cells of RPS6, RPS9, RPS17 and RPS24 but silencing of these proteins did not block p53 protein stabilization caused by actinomycin D (Figure 3D). This shows that RPL11 but not RPS9 plays a critical role in the p53 response following actinomycin D treatment.

Finally, we wanted to investigate the synthesis of new p53 protein under conditions of actinomycin D exposure or after silencing of RPS9, in the presence or absence of siRPL11. We labeled U2OS cells for 15 minutes and used p53 immunoprecipitation to monitor total and newly synthesized p53 but found no indication that RPL11 is required for p53 protein synthesis in this experimental setting (Figure 3E and F). A marginal increase in new p53 protein could be seen in actinomycin D treated cells (Figure 3E), but the interpretation of this is difficult because actinomycin D may also stabilize the pool of newly synthesized p53, and the overall levels of translation was slightly higher in this sample. The results taken all together support the notion that RPL11 predominantly controls p53 at a post-translational level and that RPL11 is not strictly required for p53 synthesis in this particular experimental setting, but an additional minor effect on p53 production is not ruled out.

### RPS9 Silencing Impairs Production of 18S rRNA and Induces p53 in Glioma Cells

**Figure 4:**
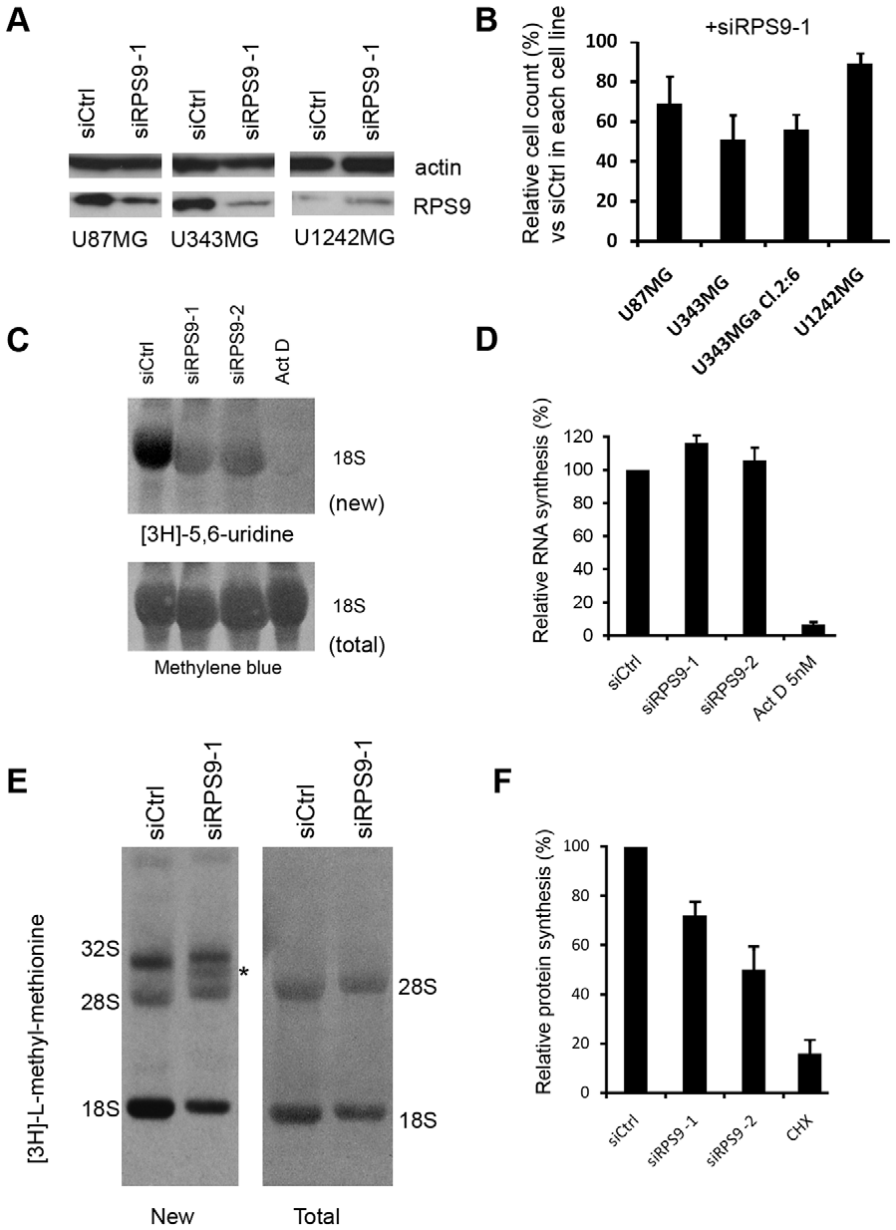
Ribosomal protein S9 is required for 18S rRNA production. (A) RPS9 knockdown efficiency in glioma cell lines U87MG, U343MG and U1242MG as determined by immunoblotting for RPS9 relative to actin. (B) Cell lines from the experiment performed in (A), and U343MGa Cl2:6 cells, were transfected with siCtrl or siRPS9-1 and after 72 hours the number of viable cells relative to siCtrl treated cells set to 100% was determined for each cell line. For this assay, equal numbers of cells were plated in triplicate at day 0, and cultures transfected on day 1, and then trypsinized and counted 72 hours later. (C) Total RNA from the experiment in (C) was separated on a 1% agarose-formaldehyde gel, transferred onto nylon membranes and subjected to autoradiography. Newly synthesized 18S rRNA is indicated, while the total level of 18S rRNA transferred to the membrane was visualized by methylene blue staining. Actinomycin D at a final concentration of 5 nM was added 3 hours prior to labeling and served as a control for inhibition of rRNA transcription. (D) U343MGa Cl2:6 cells were transfected with siRNAs as indicated and after 72 hours the cells were labeled with [^3^H]-uridine for another 2 hours. Radioactive incorporation was measured using liquid scintillation counting and expressed as cpm/µg total RNA and then normalized to the level in siCtrl transfected cells set to 100%. Results presented are averages and SEM representing three different experiments. (E) U343MGa Cl2:6 cells transfected with siRPS9-1 or siCtrl for 72 hours were incubated with [methyl-^3^H]-L-methionine for 2 hours to label the newly synthesized rRNA precursors followed by isolation of total RNA. Equal amounts of total RNA were next separated on a 1% agarose-formaldehyde gel, transferred onto nylon membranes, and subjected to autoradiography. Newly synthesized 18S rRNA is indicated (left panel) and total 28S and 18S rRNA transferred to the membrane was visualized by methylene blue staining (right panel). An asterix denotes apparent accumulation of an intermediate rRNA precursor. (F) U343MGa Cl2:6 cells transfected with siRNAs for 72 hours as indicated were labeled with [^35^S]-methionine. Cells were starved of methionine and cysteine for 30 min prior to the addition of [^35^S]-methionine. After labeling for 2 hours the protein extracts were prepared. Radioactive incorporation was measured by using liquid scintillation counting and expressed as cpm/µg total protein and then normalized to control cells set to 100%. Results presented are averages and SEM representing three different experiments. Cycloheximide (100 µg/ml) was used as a positive control to inhibit protein synthesis.

So far, most studies on ribosomal protein-p53 signaling have been carried out in U2OS cells and it remains unclear if, or to what extent, this regulatory mechanism operates in other cell types. We found that endogenous RPS9 could be efficiently silenced in U343MG and in U87MG glioma cells whereas no change in RPS9 expression was seen in U1242MG cells upon siRPS9 transfection (Figure 4A). Of note, is the already low level of RPS9 in untreated U1242MG cells. A clear reduction in the number of cells in cultures treated with siRPS9 relative to siCtrl treated cultures was seen for U87MG, U343MG, and U343MGa Cl2:6 cells but not for U1242MG (Figure 4B). As a further specificity control, we verified that the RPS9 siRNA oligonucleotide had no effect on mouse NIH3T3 cell proliferation whereas a mouse rps9 siRNA oligo efficiently inhibited the proliferation of these cells (Figure S1C). To assess the effect of RPS9 knockdown on the synthesis of mature 28S and 18S rRNA we labeled knockdown and control U343MGa Cl2:6 cells with [^3^H]-uridine for 2 hours and examined the labeled and isolated rRNA following gel electrophoresis and blotting to a nylon membrane. We found that in RPS9 knockdown cultures very little mature 18S rRNA was produced during the labeling period (Figure 4C), however overall cellular RNA synthesis continued (Figure 4D). Actinomycin D (5 nM) efficiently blocked synthesis and labeling of rRNA (Figure 4C and D). In an experiment using [^3^H]-L-methyl methionine to label newly synthesized rRNA we found that the production of 18S rRNA was clearly impaired (Figure 4E). There was a marginal decrease in the new synthesis of 28S rRNA. Moreover, the depletion of RPS9 in U343MGa Cl2:6 cells decreased the overall incorporation of [^35^S]-methionine into nascent protein by 30–50% (Figure 4F). The mechanism of maintaining rRNA synthesis at high levels despite activation of p53 remains to be determined. As suggested by others, this might be a general compensatory mechanism in response to a deficiency in ribosomes [50].

**Figure 5:**
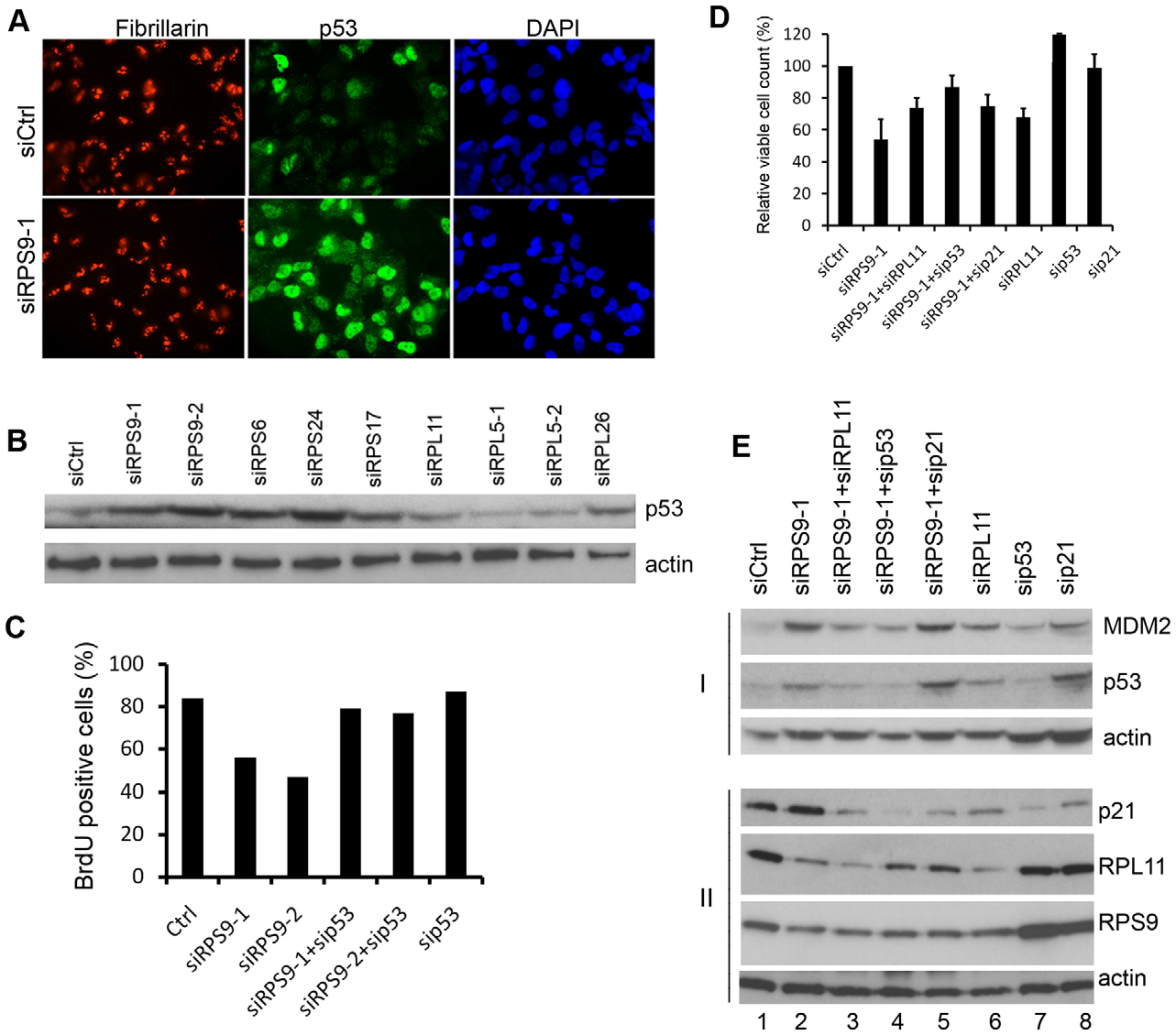
Depletion of ribosomal proteins activates p53 in glioma cells. (A) Increased nuclear expression of p53 is seen in the majority of U343MGa Cl2:6 cells after silencing of RPS9. Cells were stained for p53 and nucleolus marker fibrillarin (p53-green, fibrillarin-red, DNA-blue). (B) U343MGa Cl2:6 cells were transiently transfected with siRNAs targeting RPS9 and other ribosomal proteins, 100 nM final concentration in each case, as indicated in the figure. Cells were harvested 48 hours after transfection and levels of p53 protein determined by immunoblotting. (C) U343MGa Cl2:6 cells were transfected with siCtrl, RPS9-1, RPS9-2 or p53 siRNA as indicated. The cells were incubated with BrdU at 24 hours post transfection for another 24 hours. The cells were fixed and stained with anti-BrdU antibodies and the average of BrdU-positive cells is shown (%). (D) Knockdown of RPL11, p53 or p21 partially restores cell counts caused by the knockdown of RPS9. U343MGa Cl2:6 cells were transfected with siCtrl or with RPS9 siRNA in combination with or without RPL11, p53 or p21 siRNA and cells were counted 72 hours after transfection. Shown is the mean and SEM of three independent experiments normalized to control. (E) Co-depletion of RPL11 with siRPS9 in U343MGa Cl2:6 cells impaired the induction of p21 caused by silencing RPS9. Cells were transfected with siRNAs in combinations, or alone, as indicated in the figure and cell lysates were subjected to immunoblotting to detect the expression of actin, p53, MDM2, RPL11, RPS9 and p21.

We observed that nucleoli in RPS9 depleted U343MGa Cl2:6 cells, similar to U2OS cells, were more contrasted and phase-dense than in siCtrl treated cells (Figure S2B), and data not shown. We double stained cells that had been depleted of RPS9, for p53 and the nucleolar marker fibrillarin. An increase in p53 levels was seen in the majority of RPS9 siRNA treated U343MGa Cl2:6 cells (Figure 5A). To quantify induction of p53 in glioma cells we transfected U343MGa Cl2:6 cells (wt p53) with siRNA targeting RPS9 and other ribosomal proteins (Figure 5B). We found that RPS6, RPS17 and RPS24 silencing induced levels of p53, similar to that seen in response to depletion of RPS9 whereas no increase in p53 protein levels was seen after depleting RPL11 or RPL5. Finally, depletion of RPS9 in U343MGa Cl2:6 cells induced p53-dependent cell cycle arrest according to a BrdU incorporation assay, similar to U2OS cells (Figure 5C).

### RPS9 Silencing Promotes Morphological Differentiation of Glioma Cells

**Figure 6:**
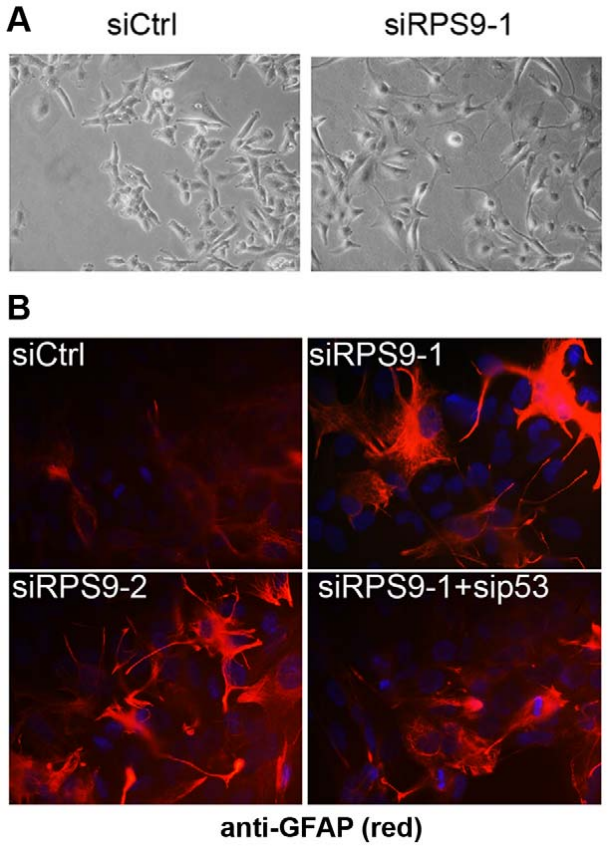
Silencing of RPS9 drives differentiation of glioma cells. (A) Phase contrast microscopy shows the morphology of U343MGa Cl2:6 cells 72 hours after transfection with siCtrl or siRPS9-1. Cells depleted of RPS9 protein show a subset of broadly bipolar cells, some of which form long cytoplasmic processes and spiking protrusions. (B) Depletion of RPS9 leads to morphological changes and accumulation of GFAP protein as evidenced by immunofluorescence staining for GFAP protein (GFAP-red, DNA-blue). These changes were reversed by co-transfecting sip53.

Visual inspection of U343MGa Cl2:6 cells under the phase contrast microscope revealed that cells depleted of RPS9 exhibited a strikingly different morphology resembling differentiation. At 48 hours, cells started to undergo a distinct morphological change characterized by the occasional formation of long and rather thin cytoplasmic processes and of round cellular bodies (Figure 6A). Using an established marker for astrocyte differentiation, glial fibrillary acidic protein (GFAP), we detected an intensified expression of GFAP in cells with spiking protrusions after depletion of RPS9 (Figure 6B). We found no evidence of cell death in U343MGa Cl2:6 cell cultures depleted of RPS9 such as an increase in the number of floating cells, or attached cells with DNA condensation. Also, senescence or appearance of DNA damage response marker protein foci following depletion of RPS9 was not observed in U343MGa Cl2:6 glioma cells (data not shown). To evaluate the impact on p53 pathway activation in U343MGa Cl2:6 cells in this setting, we depleted RPS9 alone or together with either RPL11, p53, or p21. Evidently, knockdown of RPL11, p53 or p21 partially rescued the cell proliferation defect, as measured at 72 hours of RPS9 depletion, but failed to restore cell counts to the level seen in siCtrl treated cultures (Figure 5D). SiRPS9 treated cultures had grown 54% (±11.6) whereas siRPS9+sip53 cultures had a cell count of 87% (±7.2) compared to siCtrl. Also, RPL11 depletion (only) in the long-term negatively affected cell proliferation (68%±5.0, at 72 hrs). When we co-depleted cells of RPS9 together with RPL11, p53 or p21, the morphological changes of U343MGa Cl2:6 cells were reversed (Figure S4). Similar to U2OS cells, it was found that induction of p21 was markedly impaired when co-transfecting siRNA against RPL11 (Figure 5E). Screening of other r-proteins including RPS6, RPS19, RPS17 and RPS24 revealed a knockdown phenotype similar to that of siRPS9 treated cells provided the p53 pathway was activated, suggesting that the response of U343MGa Cl2:6 cells is not unique to RPS9 loss (Figure S5).

### Induction of Cell Death in Cervical Carcinoma HeLa Cells Depleted of RPS9

**Figure 7:**
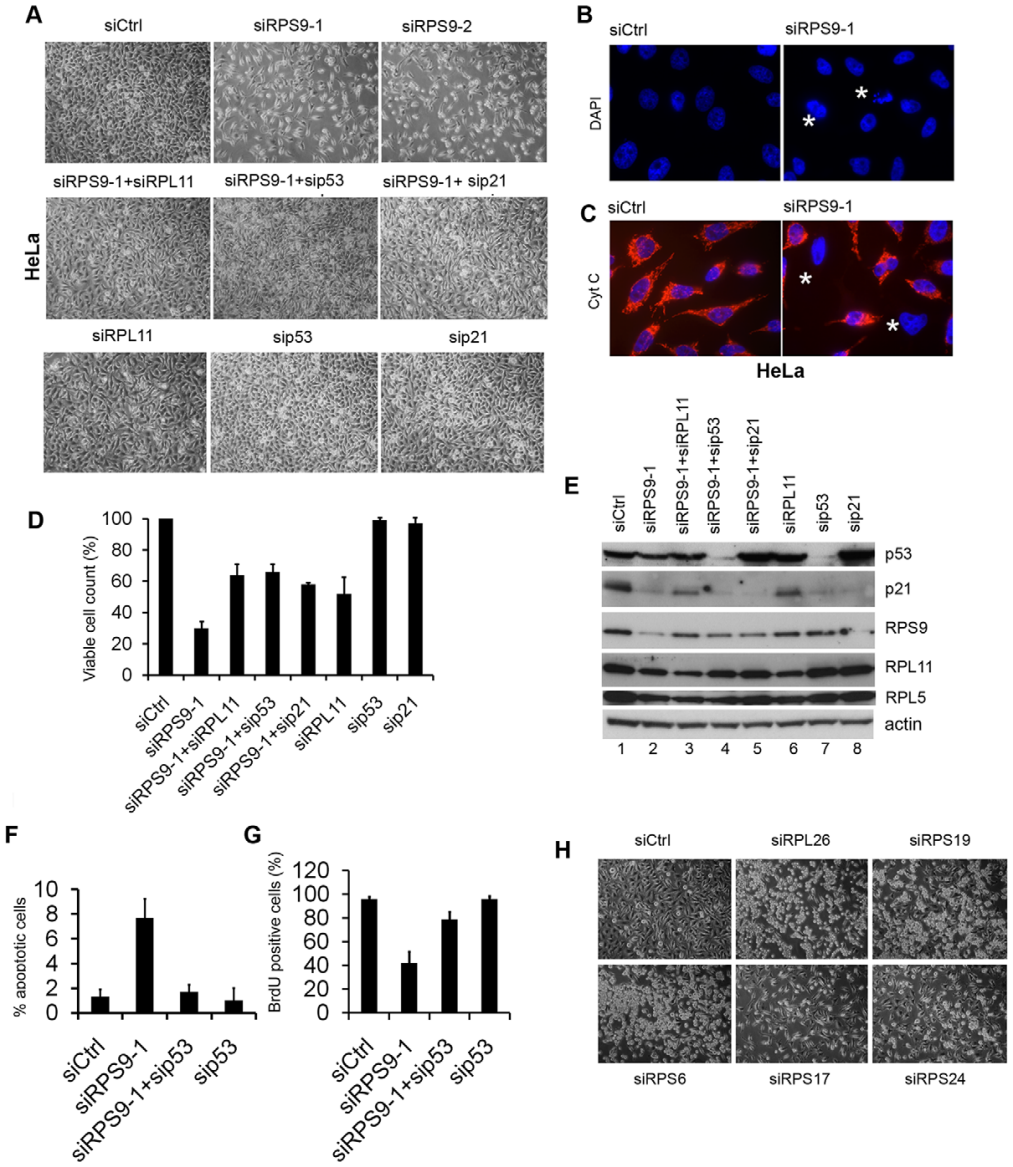
Apoptosis in HeLa cells transfected with RPS9 siRNA. (A) Photomicrographs depict the growth of HeLa cell cultures that have been depleted of RPS9. RPS9 siRNA treated cells show cell death, and few cells overall, as indicated by an increase in floating cells and a clear reduction in number of adherent cells. This effect was partially rescued by co-depletion of p53, p21 or RPL11. (B) Staining of HeLa cell nuclei using DAPI reveals the typical DNA condensation that usually occurs during apoptosis in cells depleted of RPS9 as indicated by asterix in the figure. (C) Cytochrome C is not detected in mitochondria in a fraction of HeLa cells depleted of RPS9 as indicated with an asterix (Cyt C-red, DNA-blue). No DNA condensation or cytochrome C release is seen in siCtrl treated cells. (D) Knockdown of RPL11, p21 or p53 partially restores the lower cell counts caused by the knockdown of RPS9. HeLa cells were transfected with siRPS9 alone or in combination with sip53, sip21 or siRPL11 as indicated in the figure. Cells were harvested and counted 72 hours post-transfection. Shown is mean and SEM of three independent experiments. (E) HeLa cells were transfected with indicated siRNAs for 72 hrs and expression levels of p53, p21, RPS9, RPL11 and RPL5 were analyzed by immunoblotting. A long exposure time was used for endogenous p53 detection. (F) HeLa cells were transfected with siCtrl, siRPS9, or sip53 as indicated and the number of cleaved PARP-1 product positive cells determined by immunostaining. Shown is one independent experiment in triplicate. (G) Cells were transfected with siCtrl, siRPS9, or sip53 as indicated. The cells were incubated with BrdU at 24 hours post transfection for another 24 hours and were then fixed and stained with anti-BrdU antibodies and the average of the BrdU-positive cells is shown (%). (H) HeLa cells were transfected with siRNA targeting different r-proteins as indicated in the figure.

In contrast to U343MGa Cl2:6 cells and other glioma cell lines tested, that did not undergo apoptosis, a significant number of HeLa cervical carcinoma cells showed signs of membrane blebbing, and then detached from the dish 36–48 hours after RPS9 silencing (Figure 7A). By trypan blue exclusion assay these cells were scored as not viable. DAPI staining of remaining attached cells showed frequent DNA condensation and many cells lacking cytoplasmic punctuate cytochrome C (Figure 7B and C). Silencing of other r-proteins including RPS6, RPS19, RPS17, RPS24 and RPL26 revealed a similar cellular phenotype suggesting that the apoptotic response of HeLa cells is a common response to ribosomal protein loss in this cell line (Figure 7H). To determine whether p53 pathway is activated we depleted RPS9 alone or together with either RPL11, p53, or p21. Interestingly, depletion of RPL11, p53 or p21 with siRNA could each one individually partially restore viable cell counts 72 hours post-transfection (Figure 7A and D). siRPS9 treated cells had only grown 30% (±4.3), whereas siRPS9/sip53 treated cell cultures had a cell count that was 66% (±4.9) of the control. Similar to U2OS and Cl2:6 cells there was no major change in p53 protein level in cells treated with RPS9 siRNA (Figure 7E), but we found that p21 protein was reduced in RPS9 knockdown HeLa cells, presumably as a consequence of the predominant apoptotic response. How p53 chooses to induce either apoptosis or cell cycle arrest is debated but it has been shown that in cells that are committed to p53-dependent apoptosis there is an MDM2 controlled mechanism triggering p21 degradation [51]. We next evaluated the role of p53 in siRPS9 induced HeLa cell cycle arrest and apoptosis separately. Analysis of apoptotic cells using immunodetection of cleaved PARP-1 product in adherent cells, revealed a 7% reduction in the number of dying HeLa cells when siRPS9-1 was co-transfected with sip53 (Figure 7F). In addition, twice as many HeLa cells incorporated BrdU following co-depletion of RPS9 and p53 compared to siRPS9 transfected cells only (Figure 7G). Depletion of RPL11 or RPL5 also led to inhibition of HeLa cell proliferation but this was a p53-independent effect (Figure S1E). In summary, both cell cycle arrest and apoptosis in HeLa cells have a clear p53-dependent component in the case of RPS9.

## Discussion

Outcomes of ribosomal protein loss in cultured mammalian cells and in cells *in vivo* have to date not been investigated in any greater detail but such studies have been facilitated by different conditional gene knock-out and RNA interference strategies. The drawback with RNAi methodology is the difficulty to achieve a strict 100% knockdown. Also in this setting we now know that a pre-existing pool of the ribosomal protein is present in the form of functional cytoplasmic ribosomes. These ribosomes support growth and survival of the cells, while activation of cellular checkpoints prevents further cell division if ribosome biogenesis is defective [52].

Using a set of cancer cell lines we investigated the proliferation and phenotypic response to RPS9 depletion. Silencing of RPS9 reduced the formation of new 18S rRNA. This also resulted in decreased DNA synthesis and a decline in global protein synthesis. Reduction in cell proliferation rate was due to p53 activation similar to that observed after depletion of RPS6 [30]. But silencing of RPS9 and RPL11 also resulted in p53-independent inhibition of cell proliferation in the longer run. Perhaps, an expected outcome when inhibiting the production of new ribosomes, but it cannot be ruled out that additional p53-independent checkpoints are activated. What was intriguing to us was the variety and strength of cellular responses observed following depletion of RPS9. We found that silencing of RPS9 promoted p53- and p21-dependent differentiation of U343MGa Cl2:6 glioma cells as evidenced by intensified expression of GFAP and profound changes in cell morphology. Interestingly, a very similar phenotype was seen during treatment of these cells with a histone deacetylase inhibitor trichostatin A [53]. In another study, it was found that p16INK4a expression promoted an increase in the expression of GFAP that was coupled to a stellate cell phenotype in parental U343MGa cells [54]. There are also other instances where lower expression of a ribosomal protein triggers differentiation-like responses. For example, decreased levels of RPL29 induced differentiation of LS174T colon cancer cells with increased expression of two known markers of differentiation, galectin-4 and mucin-2 [55].

Osteosarcoma cells U2OS accumulated higher levels of p21, similar to U343MGa Cl2:6 cells, but did in contrast develop a more pronounced senescence-like morphology. In fact, a fraction of the cells stained positive for SA-β-gal and DNA damage/replication stress markers. Although it has been found that loss of ribosomal protein S6 activates p53 in the absence of a detectable DNA damage response *in vivo* [7], [11], one report described an increase in p53 Ser-15 phosphorylation following depletion of WDR12, a protein involved in ribosome biogenesis [56]. It is conceivable that activation of p53 and induction of p21 in the U2OS cell line caused by RPS9 silencing may result in stalled replication forks resembling a situation of DNA replication stress.

HeLa cells showed decreased proliferation and apoptosis following depletion of ribosomal proteins. The knockdown of RPS19 also induces HeLa cell apoptosis similar to RPS9 [57]. In another study, it was demonstrated that RPS19 deficiency causes apoptosis and accelerated loss of erythroid progenitors while not affecting terminal differentiation [58]. p53 is under complete control of E6/E6AP complex rather than controlled by MDM2 in HeLa cells [59]. We would then assume that the RPL11-MDM2-p53 pathway is inactive or dormant in this cell line, but as a matter of fact, a low dose actinomycin D can reactivate p53 in HeLa cells [60]. Similarly, we found that reduced levels of RPS9 activated the p53 pathway. Induction of all p53-dependent phenotypes, senescence, differentiation and apoptosis that occurred in the respective cell cultures could be attenuated by co-depletion of RPL11.

How then is p53 activity controlled by RPL11 following RPS9 silencing? Any discussion regarding this has to start with MDM2. Regulation of MDM2 activity is complex given the abundance of many ribosomal and nucleolar proteins that can bind to MDM2, for example; p14ARF/p19ARF [61], nucleostemin[62], B23/NPM [63] and C23/nucleolin [64]. Several of these proteins could in different combinations modulate MDM2 functions after stress. One example is ribosomal protein L23 that is released from the nucleolus after actinomycin D treatment and that directly binds and inhibits MDM2 thereby activating p53 [6],[24]. Paradoxically, knockdown of RPL23 itself leads to p53 activation, but at the same time it attenuates p53 induction by actinomycin D that on its own triggers accumulation of much higher levels of p53 protein than RPL23 loss [6],[24]. It had remained a possibility that depletion of RPL11 would specifically affect the translation of *p53* mRNA leading to lower levels of p53 in a process that could depend on MDM2, or that silencing of RPL11 could lead to a global translation inhibition and thus also include *p53* mRNA. To this end, we carried out analysis of *p53* mRNA translation in cells exposed to actinomycin D, but RPL11 was not strictly required for p53 protein synthesis in this setting, although a minor effect is not ruled out.

We did not detect increased steady state levels of RPL11 in total or in NP40 soluble cellular protein extracts in RPS9 depleted cells, yet the p53 pathway was activated as evidenced by p53-dependent induction of p21. Moreover, actinomycin D led to lower levels of RPL11. Why are levels of RPL11 decreasing? One explanation is that knockdown of RPS9 causes a decline also in large subunit proteins, likely as a general cellular response to a reduced cell proliferation rate. A marginal reduction in some large subunit r-proteins as an “adaptive” response has previously been seen in other cell lines in response to RPS19 knock-down besides the more dramatic effects on r-protein levels from the same subunit [45]. Actinomycin D blocks the new synthesis of rRNA and production of ribosomes and subsequently this also leads to lower levels of RPL11. We could reproducibly see reduced levels of p53, MDM2 and in particular p21 proteins in RPL11 siRNA transfected U2OS cells also under normal conditions suggesting that RPL11 may act as a constitutive regulator of MDM2, at least in some cell types. We also found that the pool of RPL11 bound to MDM2 remained fairly constant despite decreased levels of RPL11, apparently sufficient to allow control of MDM2 and p53. Hence, a small fraction of RPL11 bound to MDM2 may set the threshold for the p53 response should stress occur. Only a complete block in new RPL11 synthesis could circumvent this control as would be the case when siRNA is used. Rapid nucleolar release of RPL11 occurring in response to actinomycin D [65], transcriptional induction of RPL11 by c-Myc [66], or translational induction of RPL11 [30] will further increase the pool of RPL11 protein that could be captured by MDM2. We presumably also have to keep in mind that the bulk of free ribosomal proteins are unstable and rapidly degraded also in the nucleus [39], [40], and that the amount of ribosomal protein bound to MDM2 represents just a minor fraction of the total cellular pool as indicated by fractionation experiments [24]. Interestingly, it has been shown that MDM2 promotes the stability of RPL11 by facilitating Nedd8 modification of RPL11 [67]. Reducing the levels of MDM2 leads to a decreased expression of RPL11 [67], something that we have observed as well. Therefore, it appears that MDM2 and a small fraction of RPL11 when bound together in a complex are both being protected from degradation, although the exact mechanism remains unclear.

While the presence of RPL11 in cells is required for maintaining the cellular levels of MDM2 and p53 in response to ribosomal/nucleolar stress, we cannot strictly rule out on basis of our data presented here, that other stress sensing molecules act upstream or in parallel with RPL11 to initiate p53 and p21-dependent responses, such as differentiation, following loss of RPS9. Moreover, p53 related or p53-independent mechanisms could also become activated. Regardless of the possible existence of other mediators of ribosome dysfunction it is striking that depletion of several r-proteins mutated in DBA (RPL11, RPL5 and RPS7) does not seem to engage the p53 pathway in different mammalian normal and cancer cell lines. However, it remains unclear to what extent the lack of p53-dependence is relevant in the pathogenesis of Diamond-Blackfan anemia since these patients frequently display mutations in RPL11 and RPL5 [17], and as we show here, RPL11 knockdown resulted in a significant inhibition of cell growth as well. From another angle, it is possible that inhibiting the expression or function of some ribosomal proteins, such as RPS9, would be one way to reinitiate differentiation processes and stop the growth of rapidly proliferating malignant tumor cells. However care must be taken, as some r-proteins are essential in regulation and induction of the cellular p53 response itself.

## Materials and Methods

### Cell Culture

Glioma cells U343MG, U1242MG, and U87MG have been previously well characterized [68], [69]. Two cell lines (U343MG and U343MGa) with different phenotypic characteristics were initially established from the same human glioblastoma multiforme biopsy. Low passage cultures of the U343MGa line have a pleomorphic appearance, whereas high passage cultures are more homogeneous, with small polygonal or short fusiform cells. This cell line, irrespective of passage level, is 100% GFAP positive. Data on clonal derivatives of the line U343MGa (Cl2:6) have been published [70]. These clones are morphologically homogeneous and retain the GFAP positive phenotype. The U343MGa Cl2:6 cell line (wt p53) was primarily selected for investigation of the effects resulting from loss of RPS9. Cervical carcinoma cells (HeLa) and osteosarcoma cells (U2OS) were purchased originally from ATCC and were mycoplasma-free. Cells were cultured in Iscoves Modified Dulbecco's medium (IMDM) containing 10% fetal bovine serum, antibiotics (100 µg of penicillin and 50 µg of streptomycin sulfate/mL), and 2 mM glutamine at 37°C, 5% CO_2_.

### Chemicals

Transcription inhibitor actinomycin D was prepared as a 1 µg/µl stock solution in DMSO and used at a final concentration of 5 nM. Nutlin-3 was prepared as a 10 mM stock in DMSO and used at a concentration of 10 µM. Cycloheximide was prepared in ethanol (100 µg/µl). All drugs were purchased from Sigma-Aldrich.

### RNA Interference

Oligofectamine was used to transfect cells in 6-well plates with siRNA, according to the manufacturer's instructions (Invitrogen). Cells were usually harvested after 72 hours unless otherwise stated. A list of siRNA oligonucleotides for human ribosomal proteins that was used is given in Table 1, and based mostly on the literature of already published and validated sequences. For human p53 siRNA the sequence was sense 5′GUAAUCUACUGGGACGGAAdTdT3′ and antisense 5′UUCCGUCCCAGUAGAUUACdCdA3′, and for human p21/CDKN1A sense 5′AGAUUUCUACCACUCCAAAdTdT3′ and antisense: 5′UUUGGAGUGGUAGAAAUCUdGdT3′. The siRNAs were purchased from Applied Biosystems and knockdown efficiencies were verified by immunoblotting.

**Table 1:** Sequences of ribosomal protein siRNA oligonucleotides used in this study.

Ribosomal protein	siRNA sequence
RPS6	Sense 5′GAAAGCCCUUAAAUAAAGAdTdT 3′
	Antisense 5′UCUUUAUUUAAGGGCUUUCdTdT 3′
RPS9-1	Sense: 5′GGAUUUCUUAGAGAGACGCdTdT 3′
	Antisense: 5′GCGUCUCUCUAAGAAAUCCdTdC 3′
RPS9-2	Sense: 5′GGAAGAAUGCCAAGAAGGGdTdT 3′
	Antisense: 5′CCCUUCUUGGCAUUCUUCCdTdC 3′
rps9-1 (mouse)	Sense: 5′ CAUCCUUCAUUGUUCGCCUdTdT 3′
	Antisense: 5′AGGCGAACAAUGAAGGAUGdGdG 3′
RPS17	Sense 5′GCGAAUUCAGAGAGGCCCAdTdT3′
	Antisense 5′UGGGCCUCUCUGAAUUCGCdTdT3′
RPS19	Sense: 5′UGGCGGCCGCAAACUGUCAdTdT 3′
	Antisense: 5′UGACAGUUUGCGGCCGCCAUC 3′
RPS24	Sense 5′GUGCCUAAGACAGAAAUUCdTdT3′
	Antisense 5′GAAUUUCUGUCUUAGGCACdTdG3′
RPL5-1	Sense: 5′GAGAGAAUCCAGUCUAUGAdTdT3′
	Antisense: 5′UCAUAGACUGGAUUCUCUCdGdT3′
RPL5-2	Sense: 5′CAGUUCUCUCAAUACAUAAdTdT3′
	Antisense: 5′UUAUGUAUUGAGAGAACUGdTdT3′
RPL11-1	Sense: 5′ GGUGCGGGAGUAUGAGUUAdTdT 3′
	Antisense: 5′UAACUCAUACUCCCGCACCdTdT 3′
RPL11-2	Sense: 5′GCAUUGGUAUCUACGGCCUdTdT3′
	Antisense: 5′AGGCCGUAGAUACCAAUGCdTdT3′
RPL26	Sense: 5′GGUUGUACGUGGACACUAUdTdT3′
	Antisense: 5′AUAGUGUCCACGUACAACCdTdG3′

### Labeling of Newly Synthesized RNA and Protein

To analyze newly synthesized rRNA, cells growing in a 6-well plate were labeled in regular medium with a final concentration of 3 µCi/ml [5,6-^3^H]uridine (Perkin Elmer) per well, for two hours. We also measured rRNA synthesis in methionine starved cells using L-[methyl-^3^H] methionine (Perkin Elmer) to a final concentration of 50µCi/ml also that for two hours. Total RNA was isolated using Trizol reagent (Invitrogen). Incorporation of radioactivity was measured using liquid scintillation counting and isolated RNA was loaded onto 1% agarose-formaldehyde gels. RNA was also blotted onto Hybond N+ membranes (Amersham) and sprayed with En^3^Hance spray (Perkin-Elmer), and subjected to fluorography for 1–3 days using Biomax MS-1 film and a Biomax transcreen LE (Perkin Elmer). To measure protein synthesis, cells were starved in methionine/cysteine free DMEM medium (Invitrogen) supplemented with dialyzed FCS (Gibco, Invitrogen) for 30 minutes, followed by a three hour pulse with 50 µCi/ml [^35^S]-methionine (Perkin Elmer). Protein extracts were prepared and total radioactive isotope incorporation was measured using liquid scintillation counting and normalized to cpm/µg total protein.

### Cell Proliferation and Cell Death Analysis

To measure proliferation, cells were seeded in 6-well plates and transfected with siRNA on the following day at around 30% confluence. Cells were then trypsinized and counted in triplicate at time points indicated, usually 72 hours after transfection. The BrdU incorporation assay was performed as in reference [62] and senescence associated β-gal assay was essentially carried out as described [71]. Cell viability was determined with trypan blue. Apoptotic cells were scored by DAPI to reveal nuclear DNA condensation complemented with analysis of PARP1 cleaved product in adherent cells using immunofluorescence with an antibody specific for cleaved PARP1 product (Cell Signaling Technology).

### Western Blotting

Cells were scraped into 0.5% NP-40 lysis buffer consisting of 0.5% Nonidet P-40, 50 mM Tris-HCl, pH 7.5, 150 mM NaCl, 50 mM NaF, 1 mM NaVO_3_, 1 mM dithiothreitol, 1× protease inhibitor cocktail (Roche). Cells were extracted for 10 minutes on ice and the lysates cleared by centrifugation. Alternatively, as indicated in the figures, an equal number of cells was collected directly into 2% SDS containing sample buffer resulting in whole cell extracts. Samples were loaded on a 10 or 12% Bis-Tris gel (Invitrogen) and blotted onto PVDF membranes. Membranes were incubated in 5% milk blocking solution with primary antibodies overnight, followed by three washes in PBS, and then incubated with secondary HRP conjugated antibody at room temperature for an additional two hours. After three washes in PBS, proteins were visualized with ECL reagent (Amersham Biosciences) or West Pico. For p53 and p21 in HeLa cells we used West Femto detection reagent (Pierce). Antibodies used in this study are listed in Table S1.

### Analysis of Newly Synthesized p53

p53 protein translation was measured in U2OS cells that were labeled with 0.4 mCi L-[^35^S]methionine and L-[^35^S]cysteine (Easy Tag EXPRESS, Perkin–Elmer) for 15 minutes, followed by immediate cell lysis in ice-cold 0.5% NP40 lysis buffer. Cell lysates containing equal amounts of proteins were immunoprecipitated with monoclonal anti-p53 antibody DO-1 (Sigma Aldrich) or IgG control overnight at 4°C, followed by one hour incubation with Protein A/G mixture beads (Amersham). After three washes of the beads in lysis buffer the immunoprecipitated material was resolved by 12% SDS-PAGE and visualized by autoradiography.

### Immunofluorescence

Cells grown and treated on coverslips in 6-well plates were washed two times in PBS, fixed using 4% PFA for 15 minutes, washed two times in PBS, permeabilized with 0.2% Triton X-100 for three minutes, washed three times in PBS, and then blocked for 30 minutes in blocking buffer (0.5% BSA in 1x PBS). Primary and secondary antibodies were diluted in blocking buffer. The primary antibodies were incubated with the cells for 60 min at room temperature followed by three washes in PBS. The secondary fluorochrome-conjugated antibodies were diluted in blocking buffer and incubated with the cells for an additional 45 min. After three PBS washes, the slides were mounted with Vectashield anti-fading agent (Vector) containing DAPI. Glass slides were analysed using a (3D) Zeiss Axioplan II microscope controlled by Axiovision 3.1 software and equipped with Plan-Apochromat 63×/1.4 and Plan-Neofluar 100×/1.30 objectives.

## Supporting Information

Figure S1 Silencing of endogenous RPS9 in U2OS. A) The use of an anti-RPS9 antibody shows a primary localization of RPS9 in nucleoli and the cytoplasm of paraformaldehyde fixed U2OS cells. The nucleolar signal is lost in most cells after silencing of RPS9. B) Efficiency of RPS9 silencing by RPS9-1 siRNA. U2OS cells were transfected to give indicated final concentrations of the siRNA and cell extracts were prepared in NP40 lysis buffer. Protein lysates were separated using SDS-PAGE and immunblotting shows the levels of RPS9, C23/nucleolin and actin. C) Silencing of mouse Rps9 using a mouse specific siRNA in NIH3T3 cells results in reduced proliferation while in contrast, human RPS9-1 siRNA was inactive in NIH3T3 cells. Cells were counted 72 hours after transfection and one representative experiment is shown. D) Relative cell counts for cells depleted of RPS9 in WI38 and WI38-E6 fibroblasts, SAOS-2 (p53 null) in comparison to U2OS (wt p53). For each cell line the relative count is established against siCtrl. E) Depletion of RPL5 and RPL11 marginally inhibits HeLa cells proliferation as determined by counting all viable cells 48 hours after transfection.
(8.77 MB TIF)

Figure S2 Nucleolar morphology in RPS9 depleted cells. U2OS cells (A) and U343MGa Cl2:6 cells (B) were transfected with siCtrl or siRPS9-1 as indicated. Cells were stained with anti-fibrillarin antibody (red) and DAPI for counterstaining of DNA.
(8.96 MB TIF)

Figure S3 Rescue of siRPS9 induced proliferative arrest in U2OS cells by RPL11, p53 or p21 co-depletion. Combined silencing of RPL11, p53 or p21 together with RPS9 partially restores cell proliferation and reverses the flat morphology phenotype of RPS9 treated U2OS cells.
(5.89 MB TIF)

Figure S4 Rescue of siRPS9 induced morphological changes in U343MGa Cl2:6 cells by RPL11, p53 or p21 co-depletion. Cells depleted of RPS9 frequently display extended cellular processes. SiCtrl treated cells form tightly packed cuboidal cells in colonies without process formation. Combined silencing of RPL11, p53 or p21 restores the parental cellular morphology.
(5.01 MB TIF)

Figure S5 Morphology of U343MGa Cl2:6 cells depleted of various ribosomal proteins as indicated.
(2.67 MB TIF)

Table S1 Antibodies used in study
(0.04 MB DOC)
