# Supplementary figures and images for: Silencing of Ribosomal Protein S9 Elicits a Multitude of Cellular Responses Inhibiting the Growth of Cancer Cells Subsequent to p53 Activation
Source: PLoS One. 2010 Mar 8;5(3):e9578. doi: 10.1371/journal.pone.0009578 (PMC2833189; doi:10.1371/journal.pone.0009578)

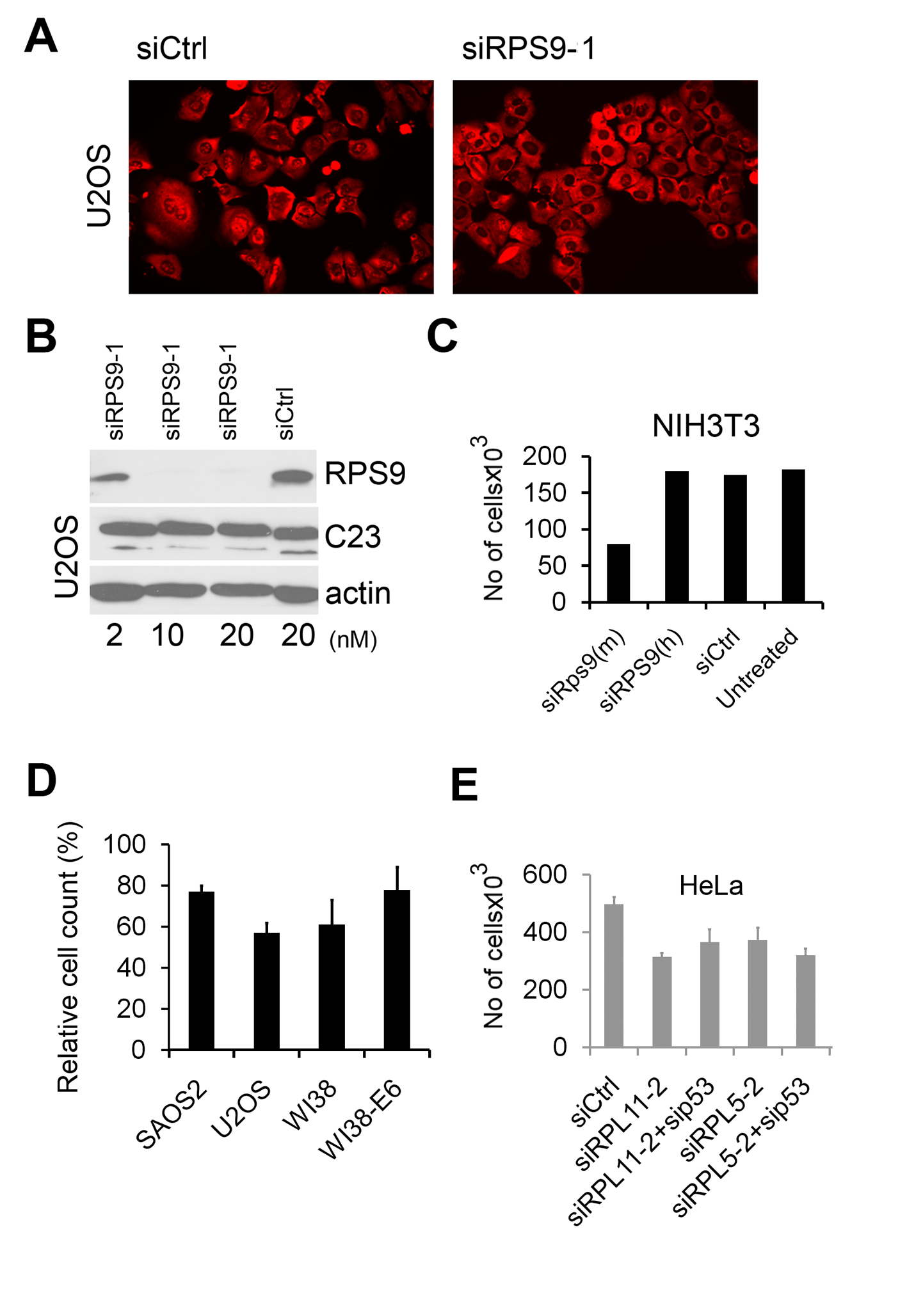

Supplement: Figure S1 — Silencing of endogenous RPS9 in U2OS. A) The use of an anti-RPS9 antibody shows a primary localization of RPS9 in nucleoli and the cytoplasm of paraformaldehyde fixed U2OS cells. The nucleolar signal is lost in most cells after silencing of RPS9. B) Efficiency of RPS9 silencing by RPS9-1 siRNA. U2OS cells were transfected to give indicated final concentrations of the siRNA and cell extracts were prepared in NP40 lysis buffer. Protein lysates were separated using SDS-PAGE and immunblotting shows the levels of RPS9, C23/nucleolin and actin. C) Silencing of mouse Rps9 using a mouse specific siRNA in NIH3T3 cells results in reduced proliferation while in contrast, human RPS9-1 siRNA was inactive in NIH3T3 cells. Cells were counted 72 hours after transfection and one representative experiment is shown. D) Relative cell counts for cells depleted of RPS9 in WI38 and WI38-E6 fibroblasts, SAOS-2 (p53 null) in comparison to U2OS (wt p53). For each cell line the relative count is established against siCtrl. E) Depletion of RPL5 and RPL11 marginally inhibits HeLa cells proliferation as determined by counting all viable cells 48 hours after transfection. (8.77 MB TIF) [file pone.0009578.s001.tif]

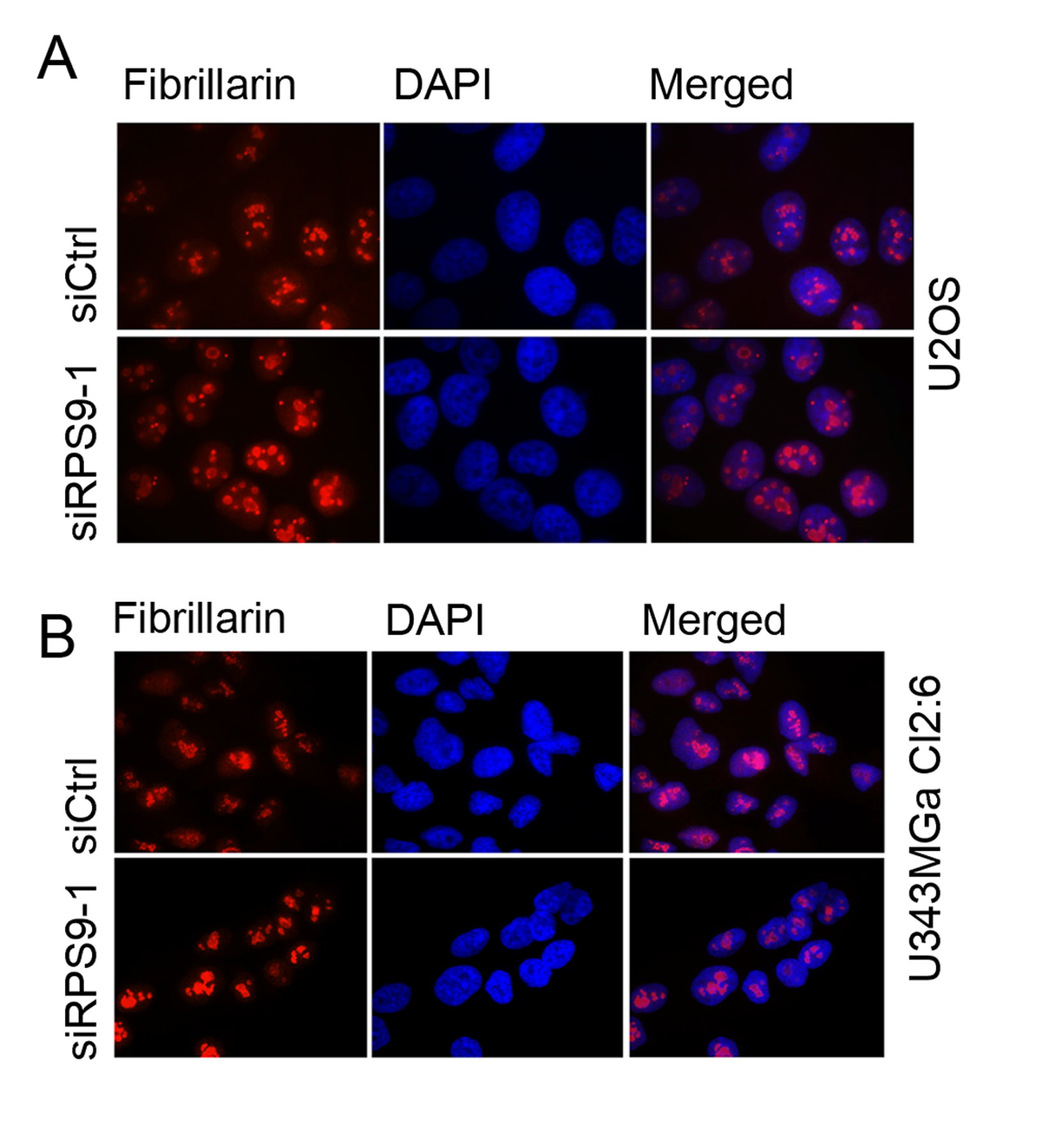

Supplement: Figure S2 — Nucleolar morphology in RPS9 depleted cells. U2OS cells (A) and U343MGa Cl2:6 cells (B) were transfected with siCtrl or siRPS9-1 as indicated. Cells were stained with anti-fibrillarin antibody (red) and DAPI for counterstaining of DNA. (8.96 MB TIF) [file pone.0009578.s002.tif]

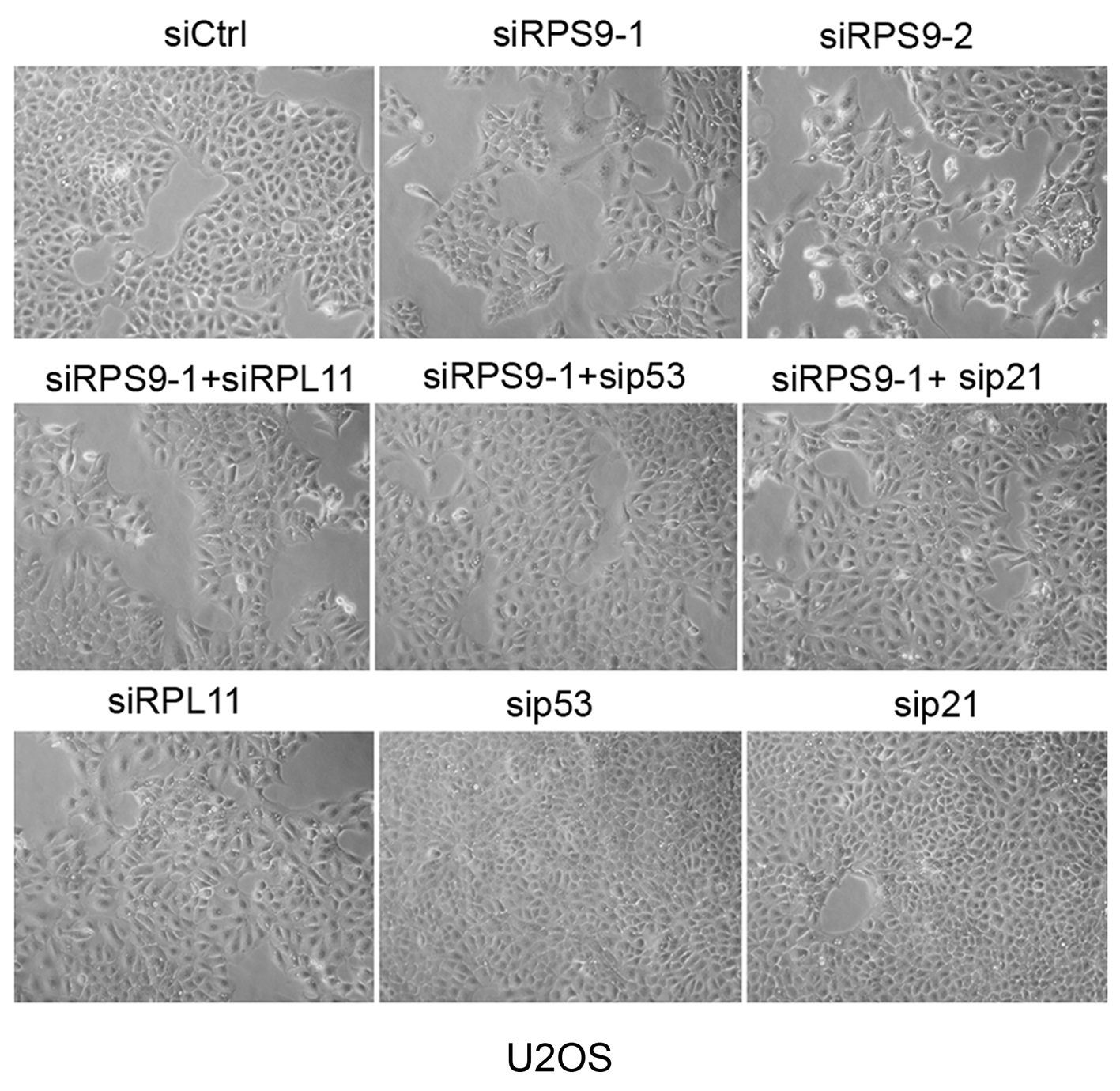

Supplement: Figure S3 — Rescue of siRPS9 induced proliferative arrest in U2OS cells by RPL11, p53 or p21 co-depletion. Combined silencing of RPL11, p53 or p21 together with RPS9 partially restores cell proliferation and reverses the flat morphology phenotype of RPS9 treated U2OS cells. (5.89 MB TIF) [file pone.0009578.s003.tif]

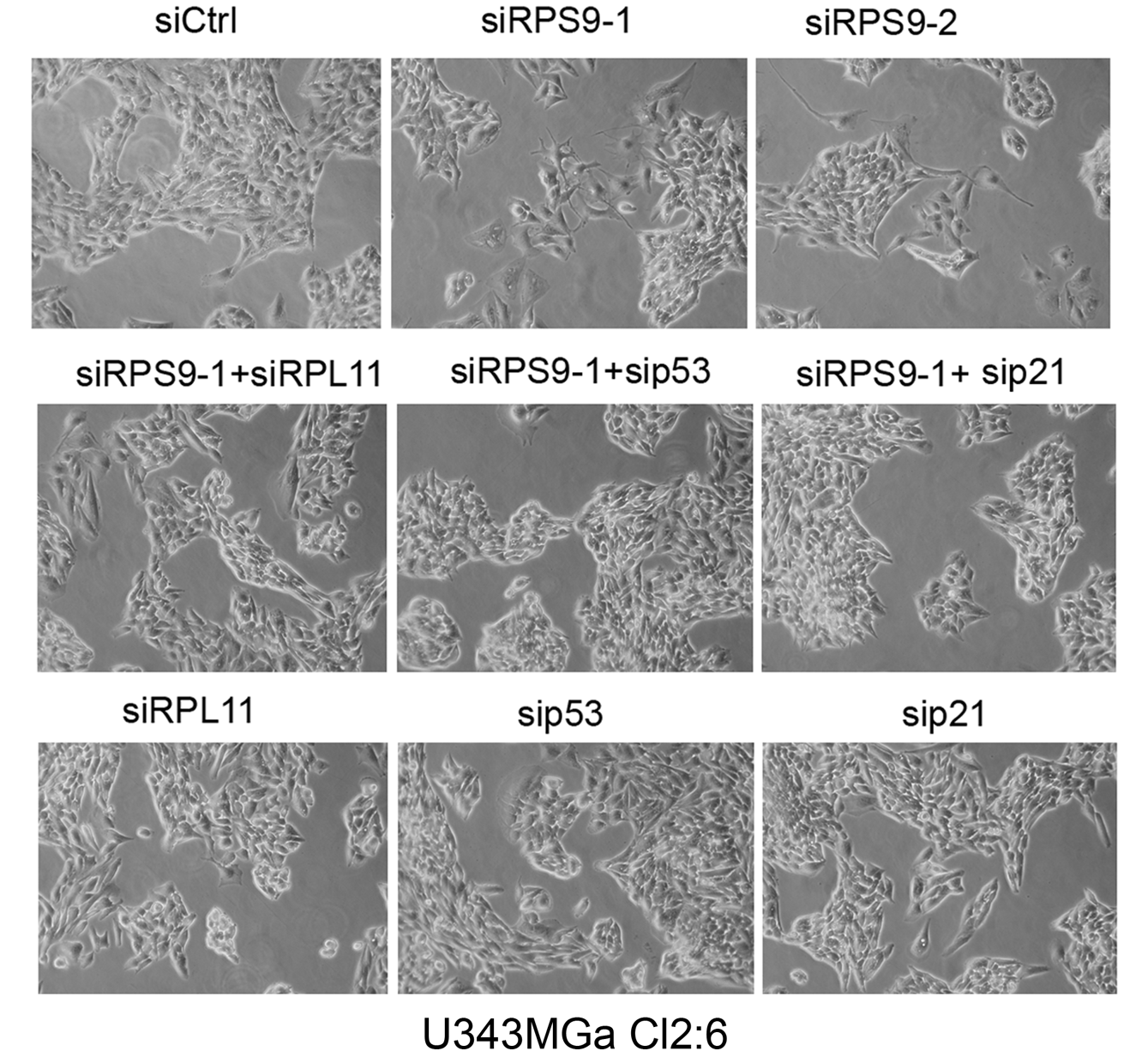

Supplement: Figure S4 — Rescue of siRPS9 induced morphological changes in U343MGa Cl2:6 cells by RPL11, p53 or p21 co-depletion. Cells depleted of RPS9 frequently display extended cellular processes. SiCtrl treated cells form tightly packed cuboidal cells in colonies without process formation. Combined silencing of RPL11, p53 or p21 restores the parental cellular morphology. (5.01 MB TIF) [file pone.0009578.s004.tif]

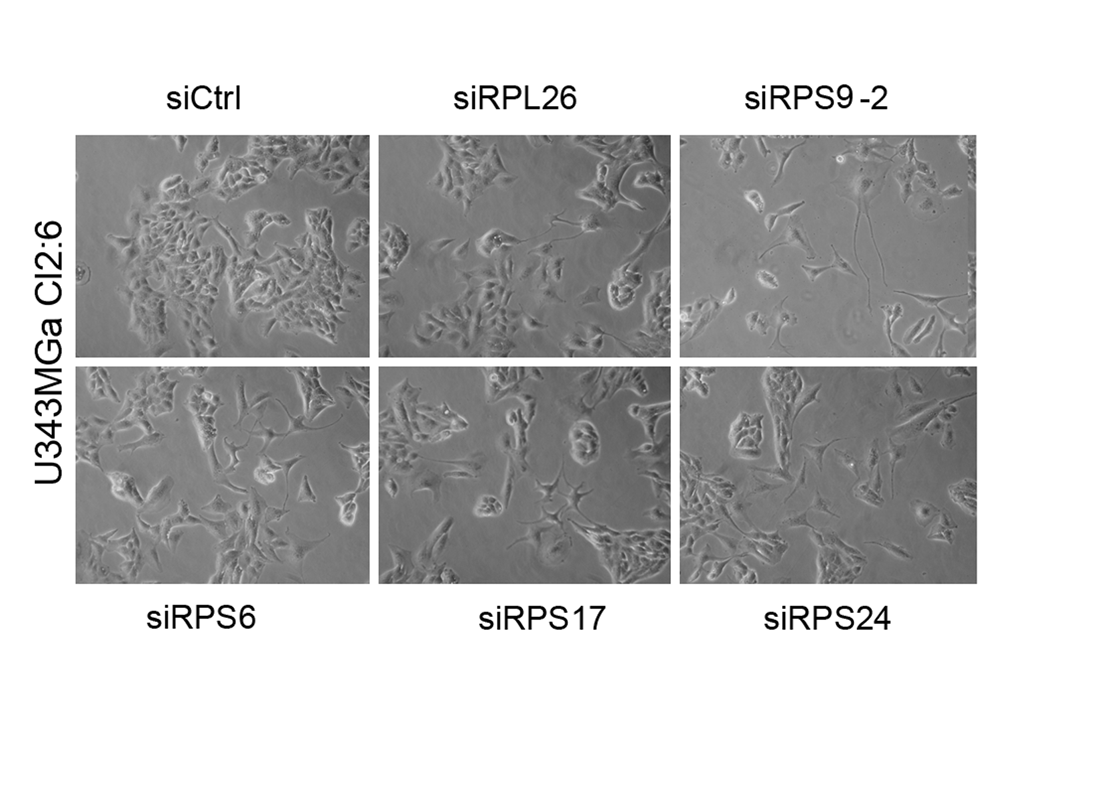

Supplement: Figure S5 — Morphology of U343MGa Cl2:6 cells depleted of various ribosomal proteins as indicated. (2.67 MB TIF) [file pone.0009578.s005.tif]
