# Supplementary material for: Silencing of Ribosomal Protein S9 Elicits a Multitude of Cellular Responses Inhibiting the Growth of Cancer Cells Subsequent to p53 Activation
Source: PLoS One. 2010 Mar 8;5(3):e9578. doi: 10.1371/journal.pone.0009578 (PMC2833189; doi:10.1371/journal.pone.0009578)
Supplement: Table S1 — Antibodies used in study (0.04 MB DOC) [file pone.0009578.s006.doc]

**TABLE S1**

**Antibodies used in study**

| **ANTIBODY** | **CLONE/CAT:#** | **COMPANY/REFERENCE** |
| --- | --- | --- |
| Actin | AC15 | Sigma Aldrich |
| Fibrillarin | Ab5821 | Abcam |
| p21 | DCS60 | Cell Signaling |
| p21 | Ab7960 | Abcam |
| p53 | FL393 | Santa Cruz |
| p53 | DO1 | Sigma Aldrich |
| EGFP | Cat# 632375 | Clontech |
| RPL5 | N37 | Ref. 26 |
| RPL26 | Cat#R0655 | Sigma Aldrich |
| RPL11 | 3A4A7 | Zymed/Invitrogen |
| RPL11 | HPA | Sigma Aldrich |
| RPS6 | 54D2 | Cell Signaling |
| RPS9 | RPS9-162 | Ref 38. |
| GFAP | Z0334 | DAKO |
| P(ser/thr)ATM/ATR | #28515 Lot 3 | Cell Signaling |
| pH2A.X(ser139) | #25775 Lot2 | Cell Signaling |
| Cytochrome C | 6H2.B4 | BD/Bioscience |
| MDM2 | N20 | Santa Cruz |
| MDM2 | SMP14 | Sigma Aldrich |
| BrdU | BRU2 | Neomarkers |
| PCNA | PC10 | Santa Cruz |
| PARP1(cleaved) | #9541 | Cell Signaling |
